# Intraorbital Wooden Foreign Body

**Published:** 2012-02-06

**Authors:** Umbareen Mahmood, Matthew Hiro, Effie Pappas-Politis, Wyatt G. Payne

**Affiliations:** Institute for Tissue Repair, Regeneration, and Rehabilitation, Bay Pines VA Healthcare System, Bay Pines, Florida, and Division of Plastic Surgery, University of South Florida, Tampa, Florida

## DESCRIPTION

A 39-year-old man presented with a 5-day history of increasing left eye swelling and erythema, after a palm tree branch struck his face.

## QUESTIONS

1. **What is the differential diagnosis of orbital cellulitis?**
2. **What is the treatment of this injury/problem?**
3. **What imaging modalities are appropriate?**

## DISCUSSION

The patient reported that he had been trimming a tree when a branch fell on his face. He was wearing sunglasses and denied direct injury to the eye itself or any open wound but noted that over the past 5 days the periocular area had become increasingly swollen. He denied impairment in vision, bleeding, drainage, fevers, photophobia, or pain, and all other reviews of systems were negative. Computed tomographic (CT) scan of the maxillofacial bones performed in the emergency department was determined to have findings suggestive of left periorbital/orbital cellulitis with early intraorbital abscess formation.

On physical examination, the patient was found to be afebrile, in no distress, with mild ecchymosis of the left malar area with three 2-mm superficial lesions of dry exudate. There was mild chemosis of the left eye and an area of nontender swelling palpated at the lateral lower eyelid and lateral malar region. Extraocular muscles, visual acuity, and light perception were intact, there was no exophthalmos or enophthalmos, eyelids were mobile, and facial bones were intact without evidence of bony stepoff. Intravenous antibiotics and topical ophthalmic antibiotics were initiated, and an ophthalmology consult for intraocular pressure and slit lamp examination was obtained; these were within normal limits. On hospital day 1, the patient had significant improvement from previous examination with a focal area of induration; with gentle manipulation, 2 cc of purulent material was expressed from the central lower eyelid laceration. The patient had immediate improvement in the lower eyelid swelling after this decompression. Operative intervention was undertaken for exploration and drainage of periorbital abscess.

Intraoperatively, initial incision resulted in the drainage of a collection of purulent fluid at the left lower eyelid. Further exploration revealed a 20-mm irregular linear wooden foreign body in the postseptal space. The area was copiously irrigated and a small drain was placed. Final bacterial culture returned positive for light growth of *Enterobacter cloacae*, *Pantoea agglomerans*, coagulase negative *Staphylococcus*, and *Escherichia hermanii*. On postoperative day 1, the patient had significant clinical improvement; the drain was removed and he was discharged home. Follow-up at 3 weeks showed complete resolution.

There is no specific symptom diagnostic for retained intraorbital foreign bodies. Common symptoms and signs include persistently red and irritated eye, diplopia, decreased visual acuity, localized pain, pressure or eyelid tightness, and disruption in ocular motility.^1^ This can result in a host of significant ocular complications including loss of vision, globe rupture, entrapment, and optic neuropathy.^2^^,^^3^ A high degree of suspicion must be maintained for the possibility in patients with a history of periocular trauma who demonstrate periorbital cellulitis, inflammation, or other ocular symptoms. Organic foreign bodies have a higher rate of sight-threatening complications and infections than nonorganic foreign bodies, and while recommendations for surgical removal may vary on the basis of the composition of the foreign body as well as their intraorbital location, appropriate broad-spectrum antibiotic treatment as well as antitetanus prophylaxis is generally accepted.^2^

Imaging studies for retained wooden and other radiopaque intraorbital foreign bodies often do not clearly assist with the diagnosis and these materials are often missed. Wooden intraorbital foreign bodies present a unique radiologic diagnostic challenge due to their varied appearance with different imaging modalities and other factors including size, shape, porosity, type, density, and especially whether the foreign body is wet or dry.^4^ While plain films are frequently performed because of their cost-effectiveness and accessibility, they may be useful only in detecting metallic intraorbital foreign bodies particularly prior to magnetic resonance imaging but are futile in detecting wood and other organic foreign bodies.^5^ Ultrasound is occasionally a helpful diagnostic adjunct due to the hyperechoic foci of wood and its acoustic shadow, but in situations of orbital trauma where there is gas in the orbit, ultrasound is insensitive in detecting wood due to interference of air.^4^ While CT is currently the imaging modality of choice for wooden intraorbital foreign bodies, numerous similar reports have demonstrated that the signal from wooden materials is often mistaken for fat or air, and that bone windows are more useful in identifying acute wooden objects than soft tissue windows. Furthermore, since several variables including size of wood, type, and wood treatments can all affect effectiveness of imaging studies, it is imperative to notify the radiologist if there is a suspicion of wooden foreign body.^6^ Interestingly, it has been documented that the Hounsfield units of a wooden foreign body increased over time as shown by follow-up CT scans, and that this may be due to the replacement of air within the wooden foreign body by fluid, hematoma, or absorption of exudates.^7^

## Figures and Tables

**Figure:**
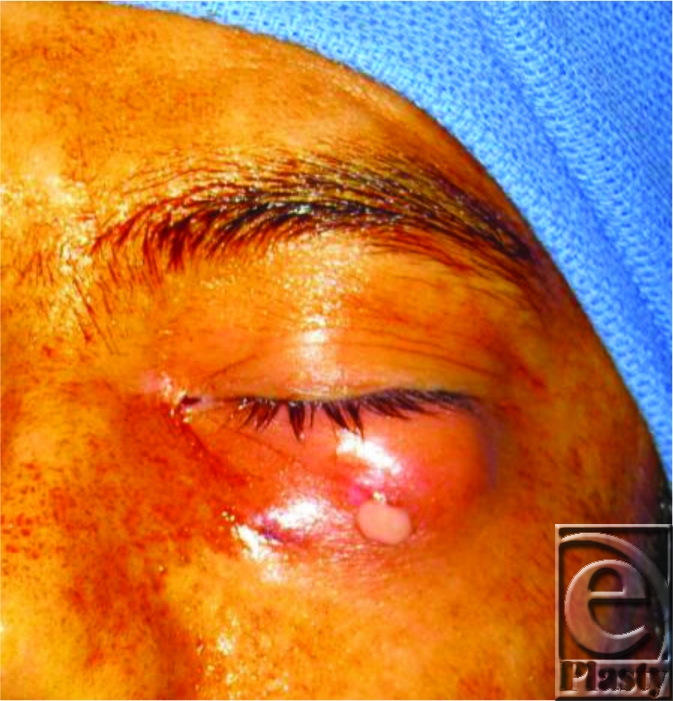


**Figure 1:**
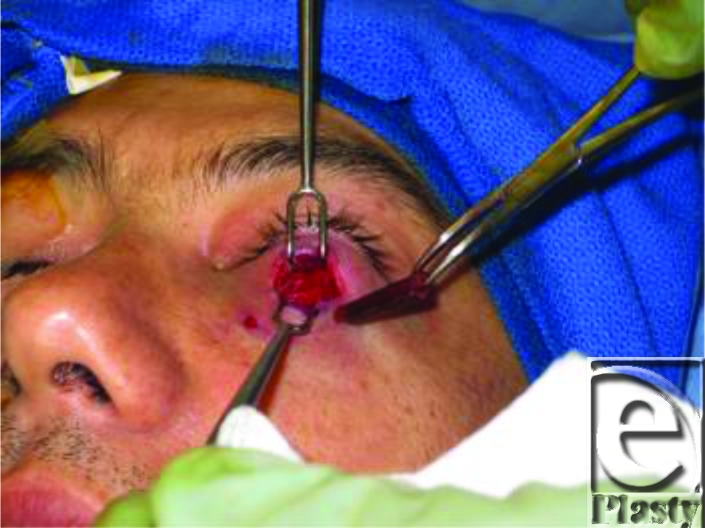
Woody foreign body removed.
